# PBPK Modeling Approach to Predict the Behavior of Drugs Cleared by Metabolism in Pregnant Subjects and Fetuses

**DOI:** 10.3390/pharmaceutics16010096

**Published:** 2024-01-10

**Authors:** Maxime Le Merdy, Ke Xu Szeto, Jeremy Perrier, Michael B. Bolger, Viera Lukacova

**Affiliations:** 1 Simulations Plus, Inc., 42505 10th Street West, Lancaster, CA 93534, USA; 2 PhinC Development, 36 Rue Victor Basch, 91300 Massy, France

**Keywords:** PBPK, pregnancy, metabolic clearance, in silico

## Abstract

This study aimed to develop a physiologically based pharmacokinetic (PBPK) model that simulates metabolically cleared compounds’ pharmacokinetics (PK) in pregnant subjects and fetuses. This model accounts for the differences in tissue sizes, blood flow rates, enzyme expression levels, plasma protein binding, and other physiological factors affecting the drugs’ PK in both the pregnant woman and the fetus. The PBPKPlus™ module in GastroPlus^®^ was used to model the PK of metoprolol, midazolam, and metronidazole for both non-pregnant and pregnant groups. For each of the three compounds, the model was first developed and validated against PK data in healthy non-pregnant volunteers and then applied to predict the PK in the pregnant groups. The model accurately described the PK in both the non-pregnant and pregnant groups and explained well the differences in the plasma concentration due to pregnancy. When available, the fetal plasma concentration, placenta, and fetal tissue concentrations were also predicted reasonably well at different stages of pregnancy. The work described the use of a PBPK approach for drug development and demonstrates the ability to predict differences in PK in pregnant subjects and fetal exposure for metabolically cleared compounds.

## 1. Introduction

Female physiology evolves during pregnancy, and understanding the impact of these changes on the PK of an active pharmaceutical ingredient (API) is critical to ensure its safety and efficacy for both the pregnant woman and the fetus. However, clinical trials rarely include pregnant women for ethical reasons [1,2], and most drugs administered during pregnancy have not been formally tested in a safe clinical environment [3]. New methods to investigate API behavior in pregnant populations have been developed, such as in vitro systems or preclinical studies [4,5], but the predictability of these tests is limited, and other approaches are mandatory. Physiologically based pharmacokinetic (PBPK) models integrate both API physicochemical information and physiological parameters that describe, in a mechanistic way, the succession of anatomical, physiological, and physical events involved in the API absorption, distribution, metabolism, and excretion (ADME) processes. Berezowska et al. reviewed the current advantages and limitations of PBPK models used to predict APIs’ PK during all stages of pregnancy [6]. The Medicines and Healthcare Products Regulatory Agency (MHRA) from the United Kingdom recently pointed out how PBPK models could support benefit–risk decisions, inform dose adjustment in pregnant populations, and investigate medicine levels in fetuses to support risk assessment [7]. However, the MHRA presented the critical need for PBPK models’ extensive qualification for high-impact applications, like clinical trial waivers [7]. In this context, our previous study focused on the distribution and elimination changes that occur during pregnancy, and presented the development of the PBPK model in GastroPlus^®^ and its validation for drugs cleared exclusively by the kidneys [8]. To further qualify the ability of the PBPK model to accurately describe the drug PK in pregnant subjects, this research project is focused on metabolic changes in pregnancy and the validation of the model using PK data for metabolically cleared drugs.

The liver is the major metabolic organ but other organs such as the intestine may play a significant role in APIs’ PK following their oral administration. Drugs can be metabolized via oxidation, reduction, hydrolysis, conjugation, or other chemical processes. For many drugs, metabolism occurs in two phases: Phase one reactions involve the formation of a new or modified functional group or molecule cleavage; Phase two reactions involve conjugation with an endogenous substance [9]. The combination of phase one and two reactions results in a more polar molecule that can be easily eliminated from the body via renal or biliary secretion. The rate of drug metabolism may vary between subjects. Indeed, for some metabolic enzymes, genetic polymorphisms have been identified and correlated with significant differences in the enzyme activities. Environmental co-factors, such as smoking, co-treatments, or some food components, can also have a significant, permanent, or temporary effect on the metabolic rate [10].

The cytochrome P450 (CYP) enzymes are the main actors of the phase one detoxification of xenobiotics [11], and the activities of a number of those (1A2, 2A6, 2B8, 2C19, 2D6, 2E1, and 3A4) have been reported to change in pregnancy [12,13]. Therefore, for APIs that are metabolized by these enzymes, the metabolic rate and plasma concentration–time course (Cp-time) are expected to change during pregnancy. However, Gong et al. reviewed the effect of gestational age and hormonal regulation on the expression of phase two enzymes in pregnant woman and the placenta. As part of this review, they identified the current knowledge gaps in phase two enzyme localization, expression, and regulation during pregnancy [14]. Due to this knowledge gap, predicting the PK in pregnant patients for APIs cleared by phase two metabolic enzymes remains a challenge.

The fetus does not significantly contribute to the maternal systemic metabolism [15]. However, the fetal metabolic capacity will appear as the liver is formed, and matures during the later stage of pregnancy. This fetal metabolism can have a significant impact on local concentrations, metabolite creation and accumulation, and potential fetal side effects.

The work presented here demonstrates the use of the PBPK model to predict both maternal and fetal exposure to APIs, mainly cleared through the metabolism. The three APIs selected for the validation are commonly used during pregnancy and almost exclusively cleared by metabolic processes: metoprolol (MET), midazolam (MID), and metronidazole (MTD).

## 2. Material and Methods

Most of the physiological changes linked with pregnancy (e.g., blood flow rates, GFR, plasma protein binding) were described in a previous publication [8]. Those changes remain important for the analysis of compounds cleared by liver metabolism. This section focuses on the maternal and fetal changes in the enzymes’ expressions level and the liver size during pregnancy.

### 2.1. Pregnancy Model

#### 2.1.1. Maternal Changes: Enzymes

Although the maternal liver volume remains constant during all stages of pregnancy, metabolic enzyme expression levels evolve as pregnancy progresses. The magnitude of changes in enzyme expression was adapted from the literature [12,13]. For the enzymes CYP2A6, CYP2B6, and CYP2E1, the expression levels only at the end of pregnancy were available, so a linear interpolation was used to calculate their expression at all gestational ages. For CYP2D6, the expressions provided in the two papers have a big discrepancy in the third trimester; therefore, the equation provided here was developed according to both datasets. For the other enzymes, the equations were either adopted from Manikandan et al. [11] or fitted with the available data in Abduljalil et al. [12] (CYP3A5 was assumed to have the same change as CYP3A4 throughout pregnancy).

$$CYP1A2\;Expression\;Level\left(\%\right)=100-3.5814\times GA+0.0495\times {GA}^{2}$$

$$CYP2A6\;Expression\;Level\left(\%\right)=100+2.05\times GA$$

CYP2B6 Expression Level (%)=100+2.25×GA

$$CYP2C19\;Expression\;Level\left(\%\right)=100-3.9083\times GA+0.0552\times {GA}^{2}$$

$$\mathrm{CYP}2D6\;Expression\;Level\;(\%)=100\;-\;0.802\times \mathrm{GA}+0.1426\times {GA}^{2}$$

$$CYP2E1\;Expression\;Level\left(\%\right)=100+2\times GA$$

$$CYP3A4\;Expression\;Level\left(\%\right)=100+2.9826\times GA-0.0741\times {GA}^{2}$$

$$CYP3A5\;Expression\;Level\left(\%\right)=100+2.9826\times GA-0.0741\times {GA}^{2}$$

In all equations, GA represents gestational age, and the expression levels are presented as percentages of the enzyme expression in a corresponding, non-pregnant female of a similar age and body weight.

#### 2.1.2. Fetal Changes: Liver Size and Enzyme Expression

A relationship linking liver tissue weight to post-menstrual age (PMA) model for newborns born 16 weeks premature (i.e., PMA = 24 weeks) up to 1-year-old infants has been previously developed [16]. For fetuses at a gestational age lower than 24 PMA, due to the lack of data, the liver tissue weight is considered to represent the same fraction of whole body weight as it does at 24 PMA, approximately 5.7% of fetal body weight (Supplementary Materials File S1).

In the current pregnancy model, the fetus is modeled as a single tissue. Therefore, the enzyme expression levels are recalculated for the whole fetal tissue using the following equation:

$${{Enz}^{i}\_expresion}^{Fetal}=\frac{FetalLiverWeight}{FetalWeight}{\;\times \;{Enz}^{i}\_expresion}^{Fetal\_liver}$$

The general equation for each enzyme is the same as that for the pediatric subject:

$${{Enz}^{i}\_expresion}^{Fetal\_liver}=\left(\frac{a_{i}\;\times \;{\left(\frac{GA}{52}\right)}^{E_{i}}}{b_{i}+{\left(\frac{GA}{52}\right)}^{E_{i}}}+PO_{i}\right)\;\times \;{{Enz}^{i}\_expresion}^{Adult\_liver}$$

where a, b, E, and PO are enzyme-specific parameters describing their ontogeny. These parameters were previously validated for neonates, infants, and children [17]. In the absence of data on the fetus, allowing their complete validation at different stages of pregnancy, they were extrapolated based on GA.

### 2.2. Model Validation Compounds

Three case studies were identified to validate the PBPK model’s ability to predict the PK in both maternal and fetal tissues at different stages of pregnancy for drugs mainly eliminated by metabolism: MET, MID, and MTD. In all cases, sufficient in vitro and in vivo information was available in the literature to develop and validate the PBPK models in healthy non-pregnant and pregnant populations. All the PBPK models were developed using GastroPlus version 9.8.3 (Simulations Plus, Inc., Lancaster, CA, USA). First, the baseline model for non-pregnant healthy adults was developed and validated for each test compound. The PBPK physiology was subsequently adapted to pregnant females using the corresponding published demographic information. The clinical trials performed in pregnant women were then predicted using the model. In all simulations, the maternal Cp-time courses as well as available fetal concentrations (e.g., fetal venous return, placenta, fetal tissue) were simulated and compared with the observed data from the corresponding clinical studies. The model was deemed acceptable if the simulated tissue concentration–time courses were within the range of the observed data, and if no systematic mispredictions could be identified. The compound properties, model settings, and clinical studies used for the model development, validation, and extrapolation to the pregnant population for all compounds are summarized in Supplementary Materials File S2.

#### 2.2.1. MET PBPK Model

The baseline distribution model consists of PBPKs with all tissues defined as perfusion-limited. The tissue/plasma partition coefficients (K_p_) were calculated using the default Lukacova method incorporating lysosomal trapping [16], as the basic pK_a_ value of 9.7 and logP value of 1.88 make MET subject to this physiological process. The MET dose used in the simulations was calculated for the free base, using the total administered dose of the salt form, the molecular weight of the free base, and the salt used in the formulations. The unbound in vivo K_m_ value (26 µM) for CYP2D6 as determined by Madani et al. from in vitro measurement of a-OH-metoprolol and O-desmethyl-metoprolol formation [18] was used in all simulations. To account for the CYP2D6 polymorphism, the default expression levels/activities for a poor metabolizer (PM), intermediate metabolizer (IM), extensive metabolizer (EM), and ultra-rapid metabolizer (UM) were used. A single V_max_ value was fitted to describe the MET Cp-time profiles for all CYP2D6 polymorphism. The renal clearance was set as plasma fraction unbound (fup) × glomerular filtration rate (GFR).

#### 2.2.2. MID PBPK Model

The baseline distribution model consists of PBPKs with all tissues defined as perfusion-limited. The Kp values were calculated using the default Lukacova method [19]. The CYP3A4 metabolism was defined using the Km and Vmax values from in vitro studies [20]. The PBPK model also includes the impact of intestinal fatty acid binding proteins (I-FABP) on the rate of MID entry into the portal vein following oral administration [21]. In all simulations for oral solutions, the stomach transit time was set to 0.1 h. The renal clearance was set to fup × GFR.

#### 2.2.3. MTD PBPK Model

The baseline PBPK models for both MTD and its metabolite hydroxy-MTD in healthy male subjects were developed with all tissues defined as perfusion-limited. The Kp values were calculated using the default Lukacova method [19]. For both MTD and hydroxy-MTD, in the absence of information from the literature, the blood-to-plasma ratio was fitted to 2.4 to accurately describe their volumes of distribution. For MTD, the in vivo K_m_ and V_max_ values for CYPs 2A6 and 3A4 were scaled from in vitro measurements obtained in rCYP systems [22]. Fitted ISEF values [23] of 4 and 13 were used for the in vitro-to-in vivo extrapolation (IVIVE) of CYP 2A6 to describe the observed data in healthy subjects obtained from two different clinical trials [24,25]. For CYP3A4, an ISEF value of 1.5 was used for the IVIVE. UGT2B7 also contributes to MTD metabolism; however, in vitro data for MTD metabolism by this enzyme were not found in the literature. Therefore, the K_m_ value for the metabolism by UGT2B7 was assumed to be equal to the in vitro value measured for CYP3A4, and V_max_ was fitted to achieve a 10% contribution of UGT2B7 to the MTD metabolism, aligned with the clinical data [26]. MTD renal clearance was set as a combination of filtration (fup × GFR) and passive secretion by adjusting the apical permeability–surface area product (PStc) in the kidney to 0.17 mL/s.

MTD biotransformation into hydroxy-MTD is catalyzed by CYPs 3A4 and 2A6, based on the published information [22]. Hydroxy-MTD metabolic elimination was mediated by UGT2B7 in the model. The same UGT2B7 Km value was used for MTD and its metabolite. Vmax was fitted using the observed concentration–time courses for hydroxy-MTD [25]. Hydroxy-MTD renal clearance was set as a combination of filtration (fup × GFR) and passive secretion by adjusting the apical PStc in the kidney to 0.015 mL/s.

## 3. Results

### 3.1. Metoprolol

MET is a cardioselective β1-blocker, which is widely used in the treatment of cardiovascular diseases. It is used to control pregnancy-induced hypertension and belongs to FDA pregnancy category C, meaning it should only be used if needed. This BCS Class I drug is cleared by CYP2D6 in the gut and liver. This metabolic enzyme is highly polymorphic, with four identified phenotypes: PM, IM, EM, and UM [27]. The plasma concentrations for compounds eliminated by CYP2D6 can be several times higher in subjects with the PM phenotype compared to the subjects with the UM phenotype. Because of this polymorphism, renal secretion contributes differently to the overall clearance based on phenotypes: in PM subjects, it can contribute up to 20% of total MET clearance following oral administration, while in EM subjects, the unchanged drug recovered in urine is less than 5% after oral administration and 10% after IV administration [28,29].

**Figure 1:**
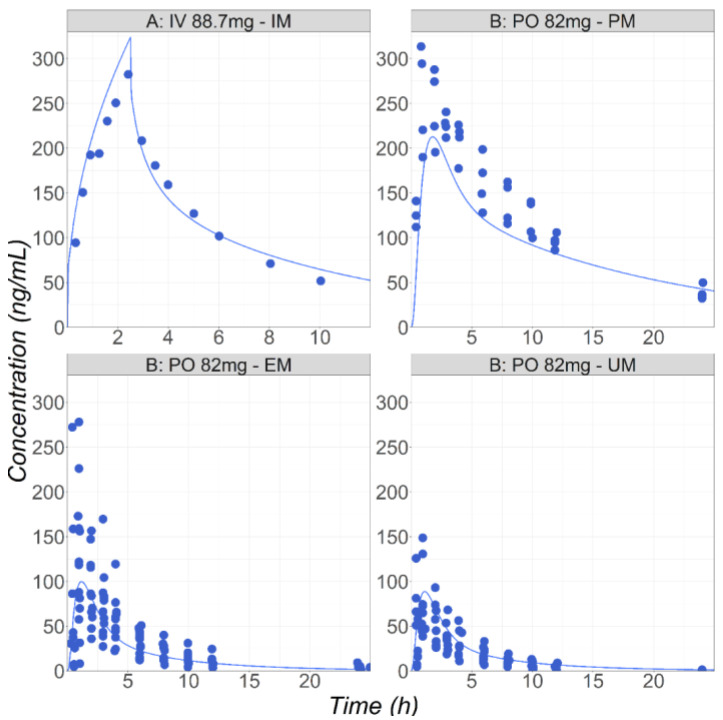
(**Study A**) Simulated and observed mean [30] Cp-time profiles after administration of metoprolol HCl in healthy fasted volunteers: 88.7 mg IV infusion for 2.5 h in male subjects (26 years old, 70 kg). (**Study B**) Simulated mean and observed individual [29] Cp-time profiles after administration of metoprolol HCl in healthy fasted volunteers (PM: 37.5 years old, 90 kg; EM: 28 years old, 77 kg; UM: 28 years old, 76 kg): 82 mg of metoprolol as an IR tablet in three groups with different CYP2D6 phenotypes: UM, EM, and PM.

To describe all the observed data in healthy subjects, a single V_max_ value (42 pmol/min/pmol CYP) was fitted and used for all the polymorph populations. The different polymorphic enzyme expressions in the gut and liver accounted for the varying rates of metabolism for the PM, IM, EM, and UM subjects. Overall, the model provides a good description the observed data following both IV and PO administration in healthy subjects [29,30] (Figure 1). Kirchheimer et al. reported separate Cp-time profiles for the PM, EM, and UM groups, and the simulations could be compared with the observed data for each phenotype [29]. For the IV administration reported by Godbillon et al., the polymorphic phenotype was not described [30]. It was found that placing this population in the IM group resulted in the best description of the observed IV data.

**Figure 2:**
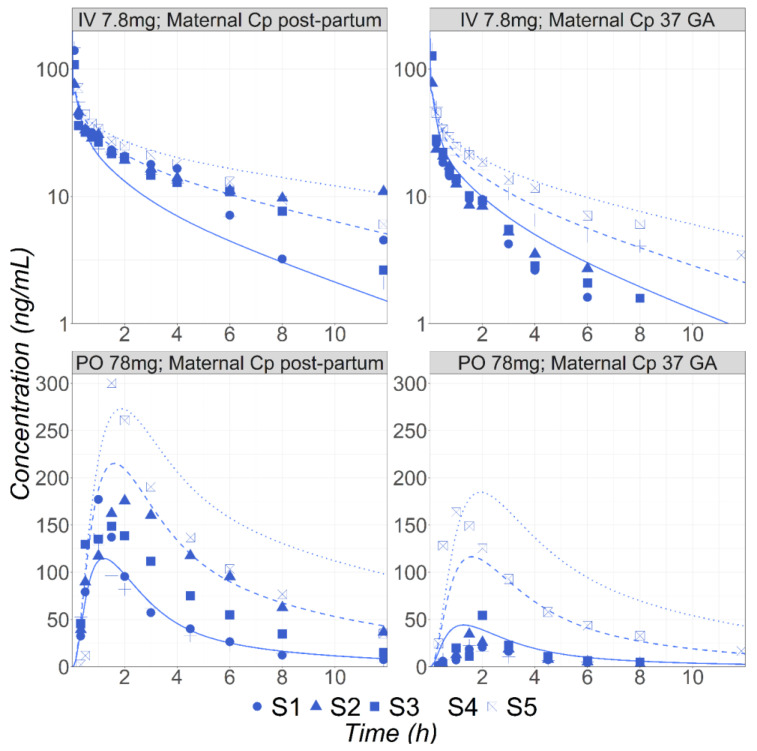
Simulated and observed [31] mean Cp-time profiles of MET in five (S1–S5) subjects either postpartum (30 years old, 72 kg) or at 37 weeks GA (30 years old, 81.8 kg) following either IV bolus (7.8 mg) or PO (78 mg) administrations. Solid, dashed, and dotted lines represent simulated concentration time courses for the EM, IM, and PM phenotypes, respectively.

After the PBPK model was validated for healthy volunteers, it was used to predict the MET PK following IV and PO administration in five subjects (labeled S1 to S5 in Figure 2) during pregnancy or postpartum [31]. Högstedt et al. did not report the subjects’ CYP2D6 phenotype but the percentage of MET excreted unchanged in the urine varies from 6.1 to 30%, suggesting the subjects included different phenotypes from PM to EM [31]. Therefore, the simulations were completed using built-in expression data for the PM, IM, and EM phenotypes. The results for the IV administration of 7.8 mg MET at 37 weeks GA and at the postpartum stage, as well as 78 mg PO administration in both groups, are presented in Figure 2. The predicted concentration ranges between the PM and EM subgroups well cover the range of the observed data for all groups of subjects, demonstrating the ability of the PBPK model to capture the effect of pregnancy on the PK of MET, an API cleared by the polymorphic CYP2D6 enzyme. It is noticeable that the fup predicted by the model for pregnant subjects based on the fup in the non-pregnant subjects (91.1% in pregnant versus 89% for postpartum subjects) accurately captured the corresponding measured values (90.8% in pregnant versus 89.1% postpartum subjects [27]).

### 3.2. Midazolam

**Figure 3:**
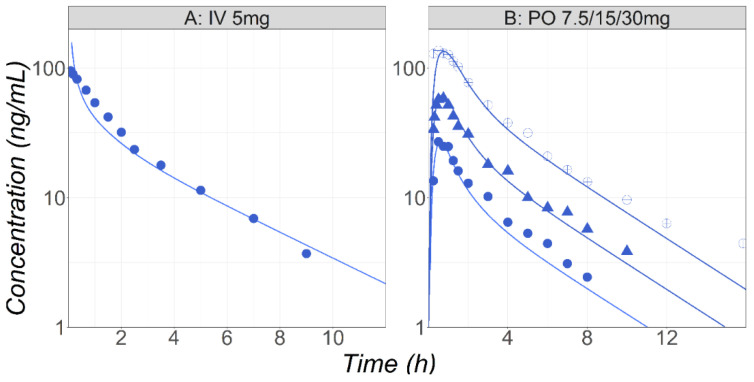
(**Study A**) Simulated and observed [33] mean Cp-time profiles after administration of MID in healthy fasted volunteers (25 years old, 70 kg) following IV bolus (5 mg) of MID. (**Study B**) Simulated and observed [34] mean Cp-time profiles after administration of either 7.5 (circles), 15 (triangles), or 30 (open circles) mg PO solution of MID in healthy fasted volunteers (38 years old, 73 kg).

MID is a benzodiazepine enhancing the effectiveness of the neurotransmitter gamma-aminobutyric acid to decrease the excitability of neurons, resulting in a calming effect on the central nervous system. In pregnant patients, MID is often used as an anesthesiologic adjuvant before or after spinal anesthesia for patients undergoing a cesarean section. MID is a BCS Class I compound. It is metabolized by the CYP3A4 enzyme into 1′-hydroxy-MID and 4′-hydroxy-MID, and then excreted primarily as a 1′-hydroxy-MID glucuronide conjugate. The unchanged amount of parent drug in the urine is less than 0.5% following IV administration [32]. In the baseline PBPK model, the MID metabolism by CYP3A4 in the gut and liver was calibrated using in vitro K_m_ and V_max_ values and the built-in enzyme expression levels in both tissues. The simulations obtained with this model are overlaid with the observed Cp-time profiles in healthy male volunteers following either IV 5 mg [33] or PO solution 7.5, 15, and 30 mg [34] administrations (Figure 3).

**Figure 4:**
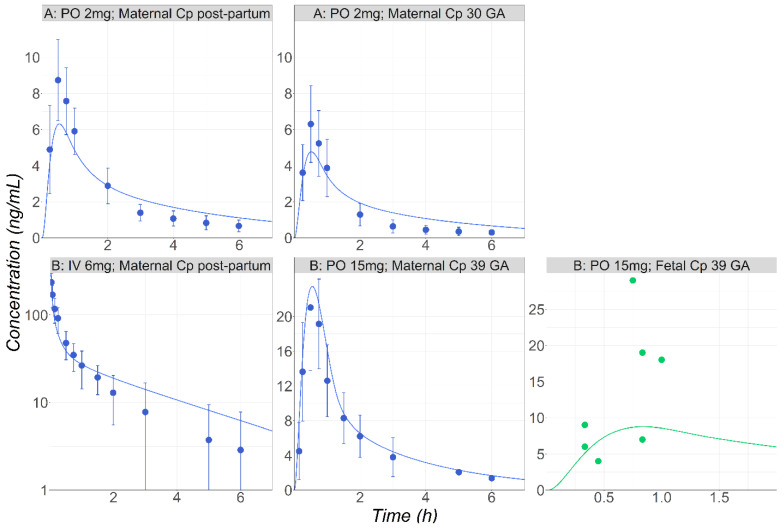
(**Study A**) Simulated and observed [35] mean Cp-time profiles after 2 mg PO tablet administration of MID in postpartum subjects (30 years old, 71.6 kg) or in pregnant subjects (30 years old, 76.4 kg) at 30 weeks GA. (**Study B**) Simulated and observed [37] mean Cp-time profiles either after IV administration of MID (0.075 mg/kg) in healthy female volunteers following delivery (30 years old, 80 kg) or following PO (15 mg) administration during pregnancy at 39 weeks GA (28 years old, 69 kg). Fetal venous return concentrations are also presented (green dots).

After the model was validated for healthy male subjects, it was applied to postpartum and pregnant female subjects receiving 2 mg PO solution of MID [35]. Initial simulations were carried out using the in vitro enzymatic parameters. Although the model was able to predict the reduced exposure in the pregnant subject versus the postpartum stage, the predicted plasma Cmax was only half of the observed value in both groups. Therefore, the in vitro Vmax (0.85 nmol/min/mg Prot.) was reduced by 17% to enhance the model predictions in the postpartum female subjects. This change may reflect the intersubject variability in the enzyme activity/expression [36]. Finally, the adjusted model was used to predict the plasma concentration–time course in the pregnant population, and it accurately described the observed data (Figure 4, study A).

The maternal plasma and fetal venous return following the single administration of 15 mg PO to the pregnant subjects were simulated [37]. The initial simulations carried out using the in vitro enzymatic parameters overpredicted the observed exposure for both the maternal and fetal plasma. Although postpartum data were not available for these subjects, the characteristics of maternal PK plasma following the IV administration of 0.075 mg/kg right after delivery to a patient group with similar population characteristics have been presented [37]. This dataset was used to scale CYP3A4 Vmax 2.47-fold. By adjusting Vmax, MID exposure following its IV administration in patients right after delivery was well described (Figure 4). The final model using the scaled Vmax could predict well both the maternal plasma and fetal venous return (Figure 4, study B).

### 3.3. Metronidazole

**Figure 5:**
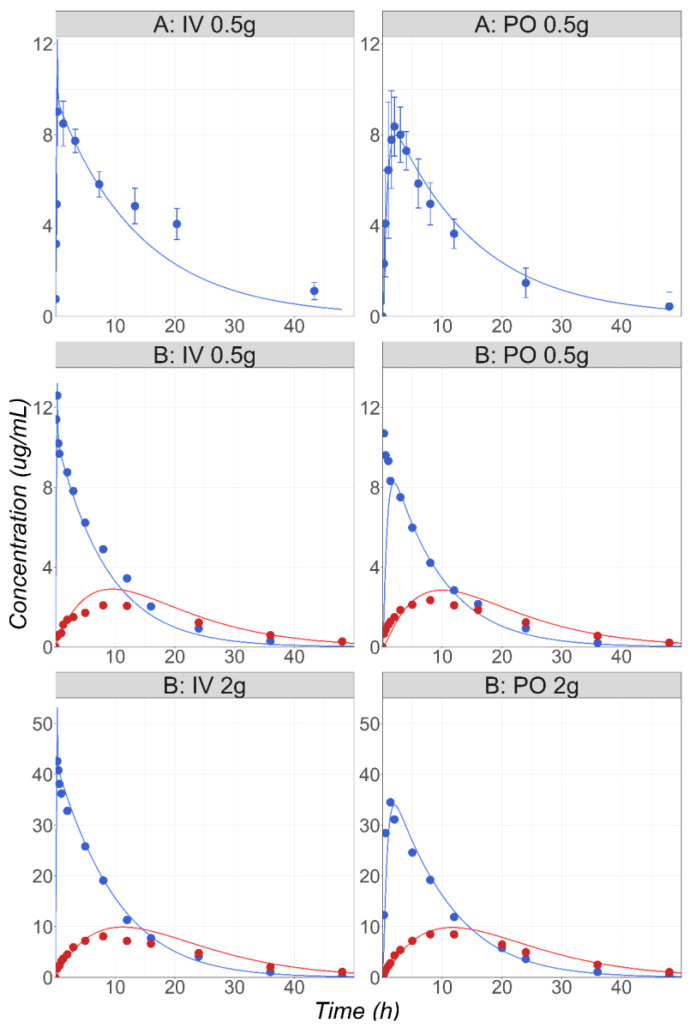
(**Study A**) Simulated and observed [24] mean MTD Cp-time profiles after administration of 0.5 g MTD IV or PO in healthy male volunteers (22 years old, 77 kg). (**Study B**) Simulated and observed [25] mean MTD (blue) and hydroxy-MTD (red) Cp-time profiles after administration of 0.5 or 2 g MTD IV or PO in healthy male volunteers (30 years old, 68 kg).

MTD is an antibiotic indicated in multiple infections caused by anaerobic bacteria. Around 80% of administered MTD is eliminated by metabolism [38] with the main contributions from the CYPs 2A6, 3A4, and UGT2B7 [22]. A fraction (20%) of the drug is excreted unchanged in the urine [38]. Cp-time courses following IV and PO administration of 0.5 g to male patients were used for the model development and validation [24]. The enzyme kinetic parameters (K_m_ and V_max_) measured in the rCYP system were obtained from the literature [22]. The in vitro Vmax was converted into the in vivo Vmax using ISEF values fitted to match the in vivo PK. The study used for the initial model development [24] required a CYP2A6 ISEF value of 4 to accurately match the observed Cp-time profile (Figure 5, study A). For external validation, the MTD PK after IV and PO administration at two dose levels (0.5 and 2 g) in healthy male subjects [25] were simulated and compared with the observed data (Figure 5, study B). To accurately capture the Cp-time profiles for MTD and its main metabolite hydroxy-MTD in this study, the ISEF value for CYP2A6 had to be increased to 13. As CYP2A6 is known to be a polymorphic enzyme [39,40], and the genotype information for the subjects involved in the studies was not reported, having a study-specific Vmax was anticipated. Both ISEF values were used to predict the MTD PK in the pregnant women.

**Figure 6:**
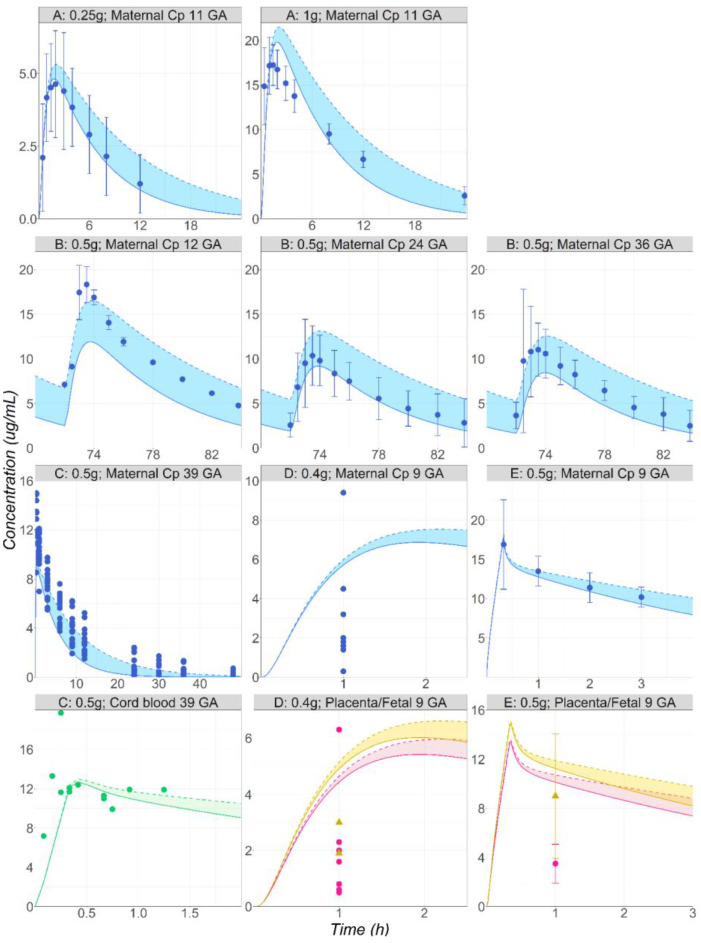
(**Study A**) Simulated and observed [41] mean Cp-time profiles after single administration of MTD to pregnant volunteers: 0.25 or 1 g PO tablet of MTD in a female pregnant subject, 11 weeks GA (29 years old, 66 kg). (**Study B**) Simulated and observed [42] mean Cp-time profiles after multiple administrations of MTD to pregnant volunteers: 0.5 g PO tablet of MTD in a female pregnant subject at either 12 (23 years old, 64 kg), 24 (23 years old, 81 kg), or 36 (23 years old, 84.7 kg) weeks GA. (**Study C**) Simulated and observed [43] individual maternal plasma (blue) and cord blood (green) concentration–time courses following the administration of 0.5 g IV infusion of MTD to female pregnant subject, 40 weeks GA (30 years old, 91 kg). (**Study D**) Simulated and observed [45] individual maternal plasma (blue), placenta (pink), and fetal tissue (yellow) concentration–time courses following the administration of 0.4 g PO tablet of MTD to female pregnant subject, 9 weeks GA (28 years old, 76 kg). (**Study E**) Simulated and observed [44] mean maternal plasma (blue), placenta (pink), and fetal tissue (yellow) concentration–time courses following the administration of 0.5 g IV infusion of MTD in a female pregnant subject, 9 weeks GA (20 years old, 58 kg).

The model developed and validated against in the vivo data from healthy non-pregnant subjects was applied to predict the PK in pregnant subjects with a variety of dosing schedules: a single 0.25 or 1 g PO dose at 11 weeks GA [41]; 0.5 g PO administered BID for four days at 12, 24, and 36 weeks GA [42]; 0.5g IV infusion at 39 weeks GA [43] or 9 GA [44]; 0.4 g single PO dose at 9 weeks GA [45].The female physiologies and GA were adapted for all datasets based on the information reported in the literature. The simulations were performed using both in vivo CYP2A6 V_max_ values determined in the simulations of the non-pregnant subjects. Overall, the range of predictions captured the in vivo data at various stages of pregnancy reasonably well (Figure 6). The maternal Cp-time profiles were predicted accurately after IV and PO administrations at different stages of pregnancy. The transplacental transport at 40 weeks GA is well captured, as the observed cord blood concentrations overlap with the predictions (Figure 6, study C [43]). The fetal tissue concentrations reported by Karhunen L. [44] at 9 weeks GA are well predicted by the model (Figure 6, study E), whereas an overprediction is obtained for the data presented in Heisterberg L.’s publication [45] (Figure 6, study D). However, it should be noted that the fetus observed data (1.9 and 3 µg/g) reported by Heisterberg L come from two patients for whom the observed maternal plasma concentrations (1.8 and 4.5 µg/mL) are also overpredicted by the model [45] (Figure 6, study D). Enhancing the maternal prediction would likely result in a better prediction of the fetal concentrations in these two subjects. Overall, for this study, both the maternal plasma and placenta predicted concentrations are within the range of observed values from this study. The placenta concentrations are overpredicted more than two-fold for the study by Karhunen L. [44] (Figure 6, study E).

In this case study, the PBPK model’s capacity to predict the maternal PK at different stages of pregnancy using a model previously validated based on a healthy non-pregnant subject population was validated. This model can also reasonably well predict the concentration in multiple fetal tissues in early and late pregnancy.

Solid lines represent the simulations obtained using the in vivo CYP2A6 Vmax calculated using an ISEF of 13. Dashed lines represent the simulations obtained using the in vivo CYP2A6 Vmax calculated using an ISEF of 4. Shaded areas represent the range of simulated concentration–time courses obtained using the in vivo CYP2A6 Vmax calculated using an ISEF > 4 and < 13.

## 4. Discussion

This study builds upon the previously developed and validated human pregnancy PBPK model. The model consists of a whole body PBPK model on the maternal side and a simplified PBPK model on the fetal side. This model was previously validated for compounds that are exclusively cleared by renal excretion, including active secretion by transporters [8]. The model structure, equations, mechanisms, and how they compare to other published PBPK models have already been discussed elsewhere [8]. In the present research, the PBPK model’s ability to predict the maternal and fetal PK for APIs that are cleared mainly by metabolism was validated using case studies for three different APIs. The maternal PK at different stages of pregnancy was predicted accurately for all studies. The fetal tissue exposures were also reasonably predicted without any additional model adjustments.

The three APIs identified to validate the pregnancy PBPK model and the enzymes’ expression level changes occurring during pregnancy are MET, MID, and MTD. The commonalities between these drugs are that 80 percent or more of the administered dose is cleared by metabolism, and the activity of all CYP enzymes contributing to the elimination of these compounds is affected during pregnancy [12,13,22,27,32]. Both the maternal postpartum and pregnancy PK data were accurately predicted for MET and MTD without any clearance model parameters adjustments (considering CYP polymorphism in the predictions). Recent PBPK models focusing on similar compounds have used a similar strategy [13,15,46,47,48,49]. Therefore, it is plausible that the relationships describing the CYP enzymes’ expression level during pregnancy are sufficient to predict the maternal PK using a PBPK model at different stages of pregnancy.

The current project focused only on phase one metabolic enzymes. Recent studies using buprenorphine and lamotrigine demonstrated that the activities of both phase one and phase two metabolic enzymes are affected by pregnancy, impacting maternal and fetal exposure to these drugs [50,51]. However, the changes in expression levels for phase two enzymes during pregnancy have not been characterized quantitatively yet [14]. As scientific knowledge evolves, further modeling studies will be performed to evaluate the ability of the pregnancy PBPK model to predict the changes in phase two metabolism in pregnancy.

Pregnancy is a dynamic process and the physiological consequences are significantly different between the first, second, and third trimesters [12,52,53,54,55]. In this project, the maternal PK was well predicted for subjects from 9 to 40 weeks GA, confirming the PBPK model’s ability to scale to all stages of pregnancy for the maternal PK using models previously validated for a non-pregnant healthy population. Based on previous work [8] and MID and MTD case studies, the fetal blood concentration is also relatively well predicted for the second and third trimesters. However, extrapolation to the first trimester will require further validation in the future. The minimal dataset of MTD in the placenta and fetal tissue indicates the exposure is relatively well described during the first trimester in those biological spaces. It is known that there are significant physiological changes during the second and third trimesters for the placenta and fetal tissues. Yet, due to the lack of observed exposure data in those tissues during the later stages of pregnancy, the PBPK model predictions could not be fully validated. Hence, those simulations should remain limited to an exploratory role until further clinical data are available.

Fetal PBPK structures can be composed of a single compartment, as the one proposed in this research, or more complex associations of different tissues [56,57,58]. The ultimate aim of the pregnancy PBPK model is to predict APIs’ behavior in the fetal tissues at different stages of pregnancy. Yet, recently published PBPK models that aimed to describe fetal exposure focused only on the fetal umbilical cord blood concentration at delivery, and solid tissue concentrations were not simulated [59,60,61,62,63,64]. In this study, the simplified fetal model structure used for the MTD case study accurately describes the fetal umbilical cord blood as well as fetal tissue concentrations. Although the observed fetal tissue data present multiple limitations due to, first, their small number; second, the lack of dynamic information regarding the concentration–time profile; and third, the absence of information on fetal tissue concentration during the second and third trimesters of pregnancy, the MTD case study provides interesting and new insights into the ability to predict fetal tissue exposure using a PBPK model. Indeed, in two studies, the predicted fetal tissue concentrations were within a reasonable range compared to the observed data, especially once the observed variability in the maternal plasma concentration was factored in. Despite these positive results, the current model structure limits its ability to predict drug PK only in the lumped fetus compartment representing solid tissues. It is expected that, as more data become available, predictions in individual fetal tissues (liver, kidney, etc.) will be possible. 

The distribution to the placental sub-compartments involved a perfusion-limited maternal and fetal placenta model for all the case studies presented in this research. The APIs’ transfer rates are therefore driven by their physicochemical characteristics, as well as the thickness of the membranes and exchange surfaces. This model structure is comparable to other structures published describing API transplacental transfer [65]. Using this model setting, the fetal umbilical cord concentration–time courses for MID and MTD were well predicted without any parameter adjustments. Similar processes were selected to model the transplacental transfer of metabolically cleared APIs in other recently published PBPK models [13,15,47,66,67]. Interestingly, for drugs that are substrates of transporters, a permeability-limited model structure for the maternal and fetal placenta tissues provided better predictions [8]. This structural change was mandatory to account for the effect of transporters, present on the placental apical membrane, on the shape of the fetal cord blood concentration–time course [54,68]. Using this model structure was a surrogate to implementing the transporters directly into the placenta tissue, as the expression levels at different stages of pregnancy remain mostly unknown [54,69]. 

The current model can describe with reasonable accuracy the API concentration in all fetal-related compartments and gives a very good prediction for the maternal plasma concentrations at different pregnancy stages. Future work will focus on adding a more detailed fetal PBPK model to assist with the prediction of local tissue concentrations and include the activity of phase two metabolic enzymes on the maternal side.

## 5. Conclusions

The work presented here has described the extended validation and use of a PBPK model to predict the changes in PK during pregnancy for compounds mainly metabolically cleared by phase one enzymes. The current approach has provided accurate maternal and reasonable fetal exposure predictions at different pregnancy stages. Although this approach is promising to predict fetal tissue exposure during the first trimester, validation is still needed for the second and third trimesters of pregnancy, and for compounds predominantly metabolized by phase two enzymes. To conclude, the ability to probe into both the maternal and fetal concentrations has made the current model useful in several other areas and explores dose predictions, drug safety, pharmacodynamics, and drug–drug interactions.

## Data Availability

The data presented in this study are available in this article (and Supplementary Materials).

## Supplementary Materials

The following supporting information can be downloaded at: https://www.mdpi.com/article/10.3390/pharmaceutics16010096/s1, File S1: Figure S1: Plot of liver weight vs. GA. Points represent experimental data, the line shows values from the fitted equation. Individual colors combined Chinese fetal liver (diamond), American fetal liver (square) and Japanese fetal liver(triangle). File S2: Table S1: clinical data used for model verification in healthy subjects as well as clinical trials used for PBPK-based predictions of pharmacokinetics in pregnant populations for metoprolol, midazolam, and metronidazole. Table S2: Key physicochemical and biopharmaceutical parameters for metoprolol used in GastroPlus simulations. Table S3: Key physicochemical and biopharmaceutical parameters for midazolam used in GastroPlus simulations. Table S4: Key physicochemical and biopharmaceutical parameters for metronidazole used in GastroPlus simulations. Table S5: Key physicochemical and biopharmaceutical parameters for hydroxy-metronidazole used in GastroPlus simulations.

## References

[1] U.S. Food and Drug Administration (2018). Pregnant Women: Scientific and Ethical Considerations for Inclusion in Clinical Trials Guidance for Industry. https://www.fda.gov/media/112195/download.

[2] European Medicines Agency (2009). Guideline on Risk Assessment of Medicinal Products on Human Reproduction and Lactation: From Data to Labelling. https://www.ema.europa.eu/en/documents/scientific-guideline/guideline-risk-assessment-medicinal-products-human-reproduction-lactation-data-labelling_en.pdf.

[3] Kappel D., Sahin L., Yao L., Thor S., Kweder S. (2023). A Comparison of FDA and EMA Pregnancy and Lactation Labeling. Clin. Pharmacol. Ther..

[4] Brent R.L. (2004). Utilization of animal studies to determine the effects and human risks of environmental toxicants (drugs, chemicals, and physical agents). Pediatrics.

[5] European Medicines Agency (2006). Guideline on the Exposure to Medicinal Products during Pregnancy: Need for Post-Authorisation Data. https://www.ema.europa.eu/en/documents/regulatory-procedural-guideline/guideline-exposure-medicinal-products-during-pregnancy-need-post-authorisation-data_en.pdf.

[6] Berezowska M., Sharma P., Pilla Reddy V., Coppola P. (2023). Physiologically Based Pharmacokinetic modelling of drugs in pregnancy: A mini-review on availability and limitations. Fundam. Clin. Pharmacol..

[7] Coppola P., Kerwash E., Cole S. (2021). Physiologically Based Pharmacokinetics Model in Pregnancy: A Regulatory Perspective on Model Evaluation. Front. Pediatr..

[8] Szeto K.X., Le Merdy M., Dupont B., Bolger M.B., Lukacova V. (2021). PBPK Modeling Approach to Predict the Behavior of Drugs Cleared by Kidney in Pregnant Subjects and Fetus. AAPS J..

[9] Almazroo O.A., Miah M.K., Venkataramanan R. (2017). Drug Metabolism in the Liver. Clin. Liver Dis..

[10] Benedetti M.S., Whomsley R., Poggesi I., Cawello W., Mathy F.-X., Delporte M.-L., Papeleu P., Watelet J.-B. (2009). Drug metabolism and pharmacokinetics. Drug Metab. Rev..

[11] Manikandan P., Nagini S. (2018). Cytochrome P450 Structure, Function and Clinical Significance: A Review. Curr. Drug Targets.

[12] Abduljalil K., Furness P., Johnson T.N., Rostami-Hodjegan A., Soltani H. (2012). Anatomical, physiological and metabolic changes with gestational age during normal pregnancy: A database for parameters required in physiologically based pharmacokinetic modelling. Clin. Pharmacokinet..

[13] Dallmann A., Ince I., Coboeken K., Eissing T., Hempel G. (2017). A Physiologically Based Pharmacokinetic Model for Pregnant Women to Predict the Pharmacokinetics of Drugs Metabolized Via Several Enzymatic Pathways. Clin. Pharmacokinet..

[14] Gong C., Bertagnolli L.N., Boulton D.W., Coppola P. (2023). A Literature Review of Changes in Phase II Drug-Metabolizing Enzyme and Drug Transporter Expression during Pregnancy. Pharmaceutics.

[15] De Sousa Mendes M., Lui G., Zheng Y., Pressiat C., Hirt D., Valade E., Bouazza N., Foissac F., Blanche S., Treluyer J.-M. (2017). A Physiologically-Based Pharmacokinetic Model to Predict Human Fetal Exposure for a Drug Metabolized by Several CYP450 Pathways. Clin. Pharmacokinet..

[16] Simulations Plus (2023). GastroPlus Manual.

[17] Johnson T.N., Rostami-Hodjegan A., Tucker G.T. (2006). Prediction of the clearance of eleven drugs and associated variability in neonates, infants and children. Clin. Pharmacokinet..

[18] Madani S., Paine M.F., Lewis L., Thummel K.E., Shen D.D. (1999). Comparison of CYP2D6 content and metoprolol oxidation between microsomes isolated from human livers and small intestines. Pharm. Res..

[19] Lukacova V., Parrott N.J., Fraczkiewicz G., Bolger M.B., Woltosz W.S. General approach to calculation of tissue:plasma partition coefficients for physiologically based pharmacokinetic (PBPK) modeling. Proceedings of the AAPS Annual Meeting.

[20] Paine M.F., Khalighi M., Fisher J.M., Shen D.D., Kunze K.L., Marsh C.L., Perkins J.D., Thummel K.E. (1997). Characterization of interintestinal and intraintestinal variations in human CYP3A-dependent metabolism. J. Pharmacol. Exp. Ther..

[21] Trevaskis N.L., Nguyen G., Scanlon M.J., Porter C.J.H. (2011). Fatty acid binding proteins: Potential chaperones of cytosolic drug transport in the enterocyte?. Pharm. Res..

[22] Pearce R.E., Cohen-Wolkowiez M., Sampson M.R., Kearns G.L. (2013). The role of human cytochrome P450 enzymes in the formation of 2-hydroxymetronidazole: CYP2A6 is the high affinity (low Km) catalyst. Drug Metab. Dispos..

[23] Proctor N.J., Tucker G.T., Rostami-Hodjegan A. (2004). Predicting drug clearance from recombinantly expressed CYPs: Intersystem extrapolation factors. Xenobiotica.

[24] Mattila J., Männistö P.T., Mäntylä R., Nykänen S., Lamminsivu U. (1983). Comparative pharmacokinetics of metronidazole and tinidazole as influenced by administration route. Antimicrob. Agents Chemother..

[25] Loft S., Døssing M., Poulsen H.E., Sonne J., Olesen K.L., Simonsen K., Andreasen P.B. (1986). Influence of dose and route of administration on disposition of metronidazole and its major metabolites. Eur. J. Clin. Pharmacol..

[26] Stambaugh J.E., Feo L.G., Manthei R.W. (1968). The isolation and identification of the urinary oxidative metabolites of metronidazole in man. J. Pharmacol. Exp. Ther..

[27] Blake C., Kharasch E., Schwab M., Nagele P. (2013). Meta-Analysis of cyp2d6 Metabolizer Phenotype and Metoprolol Pharmacokinetics. Clin. Pharmacol. Ther..

[28] U.S. Food and Drug Administration LOPRESSOR (Metoprolol Tartrate) Tablet FDA Label. https://www.accessdata.fda.gov/drugsatfda_docs/label/2008/017963s062,018704s021lbl.pdf.

[29] Kirchheiner J., Heesch C., Bauer S., Meisel C., Seringer A., Goldammer M., Tzvetkov M., Meineke I., Roots I., Brockmöller J. (2004). Impact of the ultrarapid metabolizer genotype of cytochrome P450 2D6 on metoprolol pharmacokinetics and pharmacodynamics. Clin. Pharmacol. Ther..

[30] Godbillon J., Evard D., Vidon N., Duval M., Schoeller J.P., Bernier J.J., Hirtz J. (1985). Investigation of drug absorption from the gastrointestinal tract of man. Br. J. Clin. Pharmacol..

[31] Högstedt S., Lindberg B., Peng D.R., Regårdh C.G., Rane A. (1985). Pregnancy-induced increase in metoprolol metabolism. Clin. Pharmacol. Ther..

[32] U.S. Food and Drug Administration (1984). MIdazolam Injectable Pharmacology and Toxicology Review. https://www.accessdata.fda.gov/drugsatfda_docs/nda/pre96/018654Orig1s000rev.pdf.

[33] Kupferschmidt H.H., Ha H.R., Ziegler W.H., Meier P.J., Krähenbühl S. (1995). Interaction between grapefruit juice and midazolam in humans. Clin. Pharmacol. Ther..

[34] Bornemann L.D., Min B.H., Crews T., Rees M.M., Blumenthal H.P., Colburn W.A., Patel I.H. (1985). Dose dependent pharmacokinetics of midazolam. Eur. J. Clin. Pharmacol..

[35] Hebert M.F., Easterling T.R., Kirby B., Carr D.B., Buchanan M.L., Rutherford T., Thummel K.E., Fishbein D.P., Unadkat J.D. (2008). Effects of pregnancy on CYP3A and P-glycoprotein activities as measured by disposition of midazolam and digoxin: A University of Washington specialized center of research study. Clin. Pharmacol. Ther..

[36] Inoue S., Howgate E.M., Rowland-Yeo K., Shimada T., Yamazaki H., Tucker G.T., Rostami-Hodjegan A. (2006). Prediction of in vivo drug clearance from in vitro data. II: Potential inter-ethnic differences. Xenobiotica.

[37] Kanto J., Sjövall S., Erkkola R., Himberg J.J., Kangas L. (1983). Placental transfer and maternal midazolam kinetics. Clin. Pharmacol. Ther..

[38] Houghton G.W., Thorne P.S., Smith J., Templeton R., Collier J. (1979). Comparison of the pharmacokinetics of metronidazole in healthy female volunteers following either a single oral or intravenous dose. Br. J. Clin. Pharmacol..

[39] Raunio H., Rautio A., Gullstén H., Pelkonen O. (2001). Polymorphisms of CYP2A6 and its practical consequences. Br. J. Clin. Pharmacol..

[40] López-Flores L.A., Pérez-Rubio G., Falfán-Valencia R. (2017). Distribution of polymorphic variants of CYP2A6 and their involvement in nicotine addiction. EXCLI J..

[41] Amon I., Amon K., Franke G., Mohr C. (1981). Pharmacokinetics of Metronidazole in pregnant women. Chemotherapy.

[42] Wang X., Nanovskaya T.N., Zhan Y., Abdel-Rahman S.M., Jasek M., Hankins G.D.V., Ahmed M.S. (2011). Pharmacokinetics of metronidazole in pregnant patients with bacterial vaginosis. J. Matern. Fetal. Neonatal. Med..

[43] Visser A.A., Hundt H.K. (1984). The pharmacokinetics of a single intravenous dose of metronidazole in pregnant patients. J. Antimicrob. Chemother..

[44] Karhunen M. (1984). Placental transfer of metronidazole and tinidazole in early human pregnancy after a single infusion. Br. J. Clin. Pharmacol..

[45] Heisterberg L. (1984). Placental transfer of metronidazole in the first trimester of pregnancy. J. Perinat. Med..

[46] Kammala A.K., Richardson L., Menon R. (2022). Development of physiologically based pharmacokinetic model using artificial intelligence based approaches for pravastatin during pregnancy. Am. J. Obstet. Gynecol..

[47] Darakjian L.I., Kaddoumi A. (2019). Physiologically Based Pharmacokinetic/Pharmacodynamic Model for Caffeine Disposition in Pregnancy. Mol. Pharm..

[48] Krzyzanski W., Milad M.A., Jobe A.H., Jusko W.J. (2023). Minimal physiologically-based hybrid model of pharmacokinetics in pregnant women: Application to antenatal corticosteroids. CPT Pharmacomet. Syst. Pharmacol..

[49] Zheng L., Yang H., Dallmann A., Jiang X., Wang L., Hu W. (2022). Physiologically Based Pharmacokinetic Modeling in Pregnant Women Suggests Minor Decrease in Maternal Exposure to Olanzapine. Front. Pharmacol..

[50] Zhang H., Bastian J.R., Zhao W., Chen H., Shaik I.H., Chaphekar N., Caritis S.N., Venkataramanan R. (2020). Pregnancy Alters CYP- and UGT-Mediated Metabolism of Buprenorphine. Ther. Drug Monit..

[51] Petrenaite V., Öhman I., Ekström L., Sæbye D., Hansen T.F., Tomson T., Sabers A. (2018). UGT polymorphisms and lamotrigine clearance during pregnancy. Epilepsy Res..

[52] Dallmann A., Ince I., Meyer M., Willmann S., Eissing T., Hempel G. (2017). Gestation-Specific Changes in the Anatomy and Physiology of Healthy Pregnant Women: An Extended Repository of Model Parameters for Physiologically Based Pharmacokinetic Modeling in Pregnancy. Clin. Pharmacokinet..

[53] Rasmussen K.M., Yaktine A.L., Institute of Medicine (US), National Research Council (US) (2009). Committee to Reexamine IOM Pregnancy Weight Guidelines. Weight Gain During Pregnancy: Reexamining the Guidelines.

[54] Anoshchenko O., Prasad B., Neradugomma N.K., Wang J., Mao Q., Unadkat J.D. (2020). Gestational Age-Dependent Abundance of Human Placental Transporters as Determined by Quantitative Targeted Proteomics. Drug Metab. Dispos..

[55] Carmichael S., Abrams B., Selvin S. (1997). The pattern of maternal weight gain in women with good pregnancy outcomes. Am. J. Public Health.

[56] Abduljalil K., Johnson T.N., Rostami-Hodjegan A. (2018). Fetal Physiologically-Based Pharmacokinetic Models: Systems Information on Fetal Biometry and Gross Composition. Clin. Pharmacokinet..

[57] Zhang Z., Imperial M.Z., Patilea-Vrana G.I., Wedagedera J., Gaohua L., Unadkat J.D. (2017). Development of a Novel Maternal-Fetal Physiologically Based Pharmacokinetic Model I: Insights into Factors that Determine Fetal Drug Exposure through Simulations and Sensitivity Analyses. Drug Metab. Dispos..

[58] Zhang Z., Unadkat J.D. (2017). Development of a Novel Maternal-Fetal Physiologically Based Pharmacokinetic Model II: Verification of the model for passive placental permeability drugs. Drug Metab. Dispos..

[59] Freriksen J.J.M., Schalkwijk S., Colbers A.P., Abduljalil K., Russel F.G.M., Burger D.M., Greupink R. (2020). Assessment of Maternal and Fetal Dolutegravir Exposure by Integrating Ex Vivo Placental Perfusion Data and Physiologically-Based Pharmacokinetic Modeling. Clin. Pharmacol. Ther..

[60] Mian P., Allegaert K., Conings S., Annaert P., Tibboel D., Pfister M., van Calsteren K., van den Anker J.N., Dallmann A. (2020). Integration of Placental Transfer in a Fetal-Maternal Physiologically Based Pharmacokinetic Model to Characterize Acetaminophen Exposure and Metabolic Clearance in the Fetus. Clin. Pharmacokinet..

[61] Brochot C., Casas M., Manzano-Salgado C., Zeman F.A., Schettgen T., Vrijheid M., Bois F.Y. (2019). Prediction of maternal and foetal exposures to perfluoroalkyl compounds in a Spanish birth cohort using toxicokinetic modelling. Toxicol. Appl. Pharmacol..

[62] Atoyebi S.A., Rajoli R.K.R., Adejuyigbe E., Owen A., Bolaji O., Siccardi M., Olagunju A. (2019). Using mechanistic physiologically-based pharmacokinetic models to assess prenatal drug exposure: Thalidomide versus efavirenz as case studies. Eur. J. Pharm. Sci..

[63] Anoshchenko O., Storelli F., Unadkat J.D. (2021). Successful Prediction of Human Fetal Exposure to P-Glycoprotein Substrate Drugs Using the Proteomics-Informed Relative Expression Factor Approach and PBPK Modeling and Simulation. Drug Metab. Dispos..

[64] Chen J., You X., Wu W., Guo G., Lin R., Ke M., Huang P., Lin C. (2023). Application of PBPK modeling in predicting maternal and fetal pharmacokinetics of levetiracetam during pregnancy. Eur. J. Pharm. Sci..

[65] Codaccioni M., Bois F., Brochot C. (2019). Placental transfer of xenobiotics in pregnancy physiologically-based pharmacokinetic models: Structure and data. Comput. Toxicol..

[66] Gaohua L., Abduljalil K., Jamei M., Johnson T.N., Rostami-Hodjegan A. (2012). A pregnancy physiologically based pharmacokinetic (p-PBPK) model for disposition of drugs metabolized by CYP1A2, CYP2D6 and CYP3A4. Br. J. Clin. Pharmacol..

[67] Ke A.B., Nallani S.C., Zhao P., Rostami-Hodjegan A., Unadkat J.D. (2014). Expansion of a PBPK model to predict disposition in pregnant women of drugs cleared via multiple CYP enzymes, including CYP2B6, CYP2C9 and CYP2C19. Br. J. Clin. Pharmacol..

[68] Dallmann A., Liu X.I., Burckart G.J., Anker J. (2019). van den Drug Transporters Expressed in the Human Placenta and Models for Studying Maternal-Fetal Drug Transfer. J. Clin. Pharmacol..

[69] Yamashita M., Markert U.R. (2021). Overview of Drug Transporters in Human Placenta. Int. J. Mol. Sci..

