# Supplementary material for: PBPK Modeling Approach to Predict the Behavior of Drugs Cleared by Metabolism in Pregnant Subjects and Fetuses
Source: Pharmaceutics. 2024 Jan 10;16(1):96. doi: 10.3390/pharmaceutics16010096 (PMC10820132; doi:10.3390/pharmaceutics16010096)
Supplement: Supplementary file 1 [file pharmaceutics-16-00096-s001.zip › Supplementary Materials File S1.pdf]

# *Supplementary Materials S1: Physiological parameter:*

Fetal changes: liver size

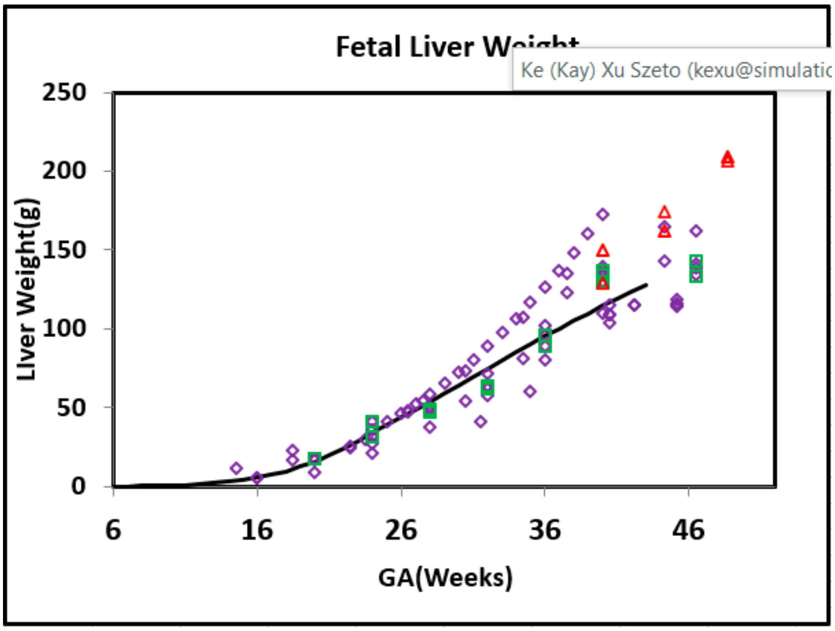


Figure S1: Plot of liver weight vs GA. Points represent experimental data (1-5), the line shows values from the fitted equation. Individual colors combined Chinese fetal liver (diamond), American fetal liver (square) and Japanese fetal liver(triangle).

# References


1. Zhang, Z.Y., Zhang, J.P., et al. (1994). "The analysis of liver weight, age and body weight in 189 fetuses." J Jining Medical College 17(3): 45.
2. Chen, T.H. (1998). "A further discussion on fetal organ growth model." Chinese J Med Phys 15(4): 217-220.
3. Hao, C., Li, W., et al. (1993). "The weight and volume of fetal viscera." Chinese J Anat 16(1): 77-80.
4. Snyder, W.S., Cook, M.J., et al. (1975). "Report of the Task Group on reference man (ICRP Publication 23)." Elsevier Science Inc., pp.480
5. Ogiu, N., Nakamura, Y., et al. (1997). "A statistical analysis of the internal organ weights of normal Japanese people." Health Phys 72(3): 368-83.
