# Supplementary material for: PBPK Modeling Approach to Predict the Behavior of Drugs Cleared by Metabolism in Pregnant Subjects and Fetuses
Source: Pharmaceutics. 2024 Jan 10;16(1):96. doi: 10.3390/pharmaceutics16010096 (PMC10820132; doi:10.3390/pharmaceutics16010096)
Supplement: Supplementary file 1 [file pharmaceutics-16-00096-s001.zip › Supplementary Materials File S2.pdf]

Supplementary Material S2: Model parameters and data origins:

Table S1 summarizes the available clinical data used for model development and validation in healthy subjects as well as clinical trials used for in silico predictions of pharmacokinetics in pregnant populations:

Table S1: clinical data used for model verification in healthy subjects as well as clinical trials used for PBPK-based predictions of pharmacokinetics in pregnant populations for metoprolol, midazolam, and metronidazole.

Dose	Administration Route	Population	Age* (years)	BW* (Kg)	Source
			**Metoprolol**		
		Baseline Model			
88.7 mg	IV inf 150'	Healthy nonpregnant	*23-29*	-	(1)
78 mg	PO	Healthy nonpregnant	*37.5 (PM)*<br>*28 (EM)*<br>*28 (UM*	*90 (PM)*<br>*77 (EM)*<br>*76 (UM*	(2)
		Pregnancy model			
7.8/78	IV/PO	Post-Partum	-	*72*	(3)
7.8/78	IV/PO	Pregnant	-	*81.8*	(3)
			**Midazolam**		
		Baseline Model			
5 mg	IV	Healthy nonpregnant	25	70	(4)
7.5/15/30 mg	PO Solution	Healthy nonpregnant	38	73	(5)
		Pregnancy model			
2 mg	PO	Post-Partum	30	71.6	(6)
2 mg	PO	Pregnant	30	76.4	(6)
0.075mg	IV	Post-Partum	30	80	(7)
15 mg	PO	Pregnant	28	69	(7)
			**Metronidazole**		
		Baseline Model			
0.5 g	IV inf 20'/PO	Healthy nonpregnant	22	77	(8)
0.5/2 g	IV inf 20'/PO	Healthy nonpregnant	30	68	(9)
		Pregnancy model			

0.25/1 g	PO	Pregnant	29	66	(10)
0.5 g BID	PO	Pregnant	23	64 (12 GA)<br>81 (24 GA)<br>84.7 (36 GA)	(11)
0.5 g	IV inf 20'	Pregnant	-	-	(12)
0.4 g	PO	Pregnant	28	-	(13)
0.5 g	PO	Pregnant	20	58	(14)

*When the demographic information where not available in the publication, default GastroPlus values were used

For all compounds, the distribution in all tissues is simulated using perfusion limited tissue models. The drug specific parameters (e.g., Log P and pKa) used as input data and their references for MET, MID and MTD and hydroxy-MTD are summarized in Table S2 to Table 5.

Table S2: Key physicochemical and biopharmaceutical parameters for metoprolol used in GastroPlus simulations.

**Parameter**	**Value**	**Reference**
logP	1.88	(15)
Diffusion coefficient	0.74 $x10^{-5}$ cm^2/s	ADMET Predictor[a]
pKa	9.7 (base)	(16)
Reference solubility	11.1 mg/mL @ pH = 9	ADMET Predictor
Solubility Factor	17.11	ADMET Predictor
FaSSIF solubility	5.98 mg/mL	ADMET Predictor
FeSSIF solubility	14.64 mg/mL	ADMET Predictor
Human effective permeability (P_{eff})	1.34 x 10^{-4} cm/s	(17)
Particle radius	25 µm	GastroPlus default
Precipitate radius	1 µm	GastroPlus default
Drug particle density	1.2 g/mL	GastroPlus default
Mean precipitation time	900 s	GastroPlus default
Blood:plasma concentration ratio (R_{bp})	1.13	(16)
Percent unbound in plasma (Fup)	89 %	(3)
Adjusted Fup	88.8 %	GastroPlus algorithm[b]
<u>Metabolism</u>		
CYP 2D6 Km,u	26 µM	(18)
CYP 2D6 Vmax	42 pmol/min/pmol CYP	Fitted

[a] Predicted using ADMET Predictor® v10.0

[b] Adjusted Fup was calculated from experimental Fup and logD @ pH = 7.4 using the default GastroPlus equation

Table S3: Key physicochemical and biopharmaceutical parameters for midazolam used in GastroPlus simulations.

**Parameter**	**Value**	**Reference**
logP	2.7	(19)
Diffusion coefficient	0.75 x10^{-5} cm^2/s	ADMET Predictor[a]
pKa	6.04 / 0.84 (base)	(20)
Reference solubility	0.054 mg/mL @ pH = 9.5	(20)
Solubility Factor	370	ADMET Predictor
FaSSIF solubility	0.11 mg/mL	Internal data
Human effective permeability (P_{eff})	3.82 x 10^{-4} cm/s	(21)
Particle radius	25 µm	GastroPlus default
Precipitate radius	1 µm	GastroPlus default
Drug particle density	1.2 g/mL	GastroPlus default
Mean precipitation time	900 s	GastroPlus default
Blood:plasma concentration ratio (R_{bp})	0.55	(22)
Percent unbound in plasma (Fup)	4.4 %	(23)
Adjusted Fup	3.9 %	GastroPlus algorithm[b]
Percent unbound in enterocytes (Fue)	4.4 %	Fitted
<u>Metabolism</u>		
CYP 3A4 Km,u	3.7 µM	(24)
CYP 3A4 Vmax	0.85 nmol/min/mg-protein	(24)
Scaling factor $Vmax_{Herbert}$	0.83	Fitted
Scaling factor $Vmax_{Kanto}$	2.47	Fitted

[a] Predicted using ADMET Predictor v10.0

[b] Adjusted Fup was calculated from experimental Fup and logD @ pH = 7.4 using the default GastroPlus equation

Table S4: Key physicochemical and biopharmaceutical parameters for metronidazole used in GastroPlus simulations.

**Parameter**	**Value**	**Reference**
logP	-0.02	(25)
Diffusion coefficient	1.11x10^{-5} cm^2/s	ADMET Predictor[a]
pKa	2.59 (base)	(26)
Reference solubility	9.5 mg/mL @ pH = 7.66	(26)
Solubility Factor	28.85	(26)
FaSSIF solubility	20.4 mg/mL	ADMET Predictor
FeSSIF solubility	16.7 mg/mL	ADMET Predictor
Human effective permeability (P_{eff})	2.54x 10^{-4} cm/s	ADMET Predictor
Particle radius	25 μm	GastroPlus default
Precipitate radius	1 μm	GastroPlus default
Drug particle density	1.2 g/mL	GastroPlus default
Mean precipitation time	900 s	GastroPlus default
Blood:plasma concentration ratio (R_{bp})	2.4	Fitted
Percent unbound in plasma (Fup)	96%	(27)
Adjusted Fup	95.5 %	GastroPlus algorithm[b]
Kidney apical Pstc	0.17 mL/s	Fitted
Metabolism		
CYP3A4 Km,u	5.38 mM	(28)
CYP3A4 Vmax	2.49 nmol/min/nmol CYP	(28)
ISEF CYP3A4	1.5	Fitted
CYP2A6 Km,u	0.289 mM	(28)
CYP2A6 Vmax	0.65 nmol/min/nmol CYP	(28)
ISEF CYP2A6	4 / 13	Fitted
UGT2B7 Km,u	5.38 mM	assumed
UGT2B7 Vmax	21.5 nmol/min/nmol CYP	Fitted

[a] Predicted using ADMET Predictor v10.3

[b] Adjusted Fup was calculated from experimental Fup and logD @ pH = 7.4 using the default GastroPlus equation

Table S5: Key physicochemical and biopharmaceutical parameters for hydroxy-metronidazole used in GastroPlus simulations.

**Parameter**	**Value**	**Reference**
logP	-0.95	ADMET Predictor[a]
Diffusion coefficient	1.109x10^{-5} cm^2/s	ADMET Predictor[a]
pKa	2.01 (base)	ADMET Predictor[a]
Reference solubility	24.23 mg/mL @ pH = 7.58	ADMET Predictor[a]
Solubility Factor	7.63	ADMET Predictor[a]
FaSSIF solubility	48.68 mg/mL	ADMET Predictor
FeSSIF solubility	26.02 mg/mL	ADMET Predictor
Human effective permeability (P_{eff})	1.41x 10^{-4} cm/s	ADMET Predictor
Particle radius	25 µm	GastroPlus default
Precipitate radius	1 µm	GastroPlus default
Drug particle density	1.2 g/mL	GastroPlus default
Mean precipitation time	900 s	GastroPlus default
Blood:plasma concentration ratio (R_{bp})	2.4	Fitted
Percent unbound in plasma (Fup)	96%	ADMET Predictor
Adjusted Fup	95.9 %	GastroPlus algorithm[b]
Kidney apical Pstc	0.015 mL/s	Fitted
Metabolism		
UGT2B7 Km,u	5.38 mM	assumed
UGT2B7 Vmax	0.2 nmol/min/nmol CYP	Fitted

[a] Predicted using ADMET Predictor v10.3

[b] Adjusted Fup was calculated from experimental Fup and logD @ pH = 7.4 using the default GastroPlus equation

References

1. Godbillon J, Evard D, Vidon N, Duval M, Schoeller JP, Bernier JJ, et al. Investigation of drug absorption from the gastrointestinal tract of man. Br J Clin Pharmacol. 1985;19(Suppl 2):113S-118S.

2. Kirchheiner J, Heesch C, Bauer S, Meisel C, Seringer A, Goldammer M, et al. Impact of the ultrarapid metabolizer genotype of cytochrome P450 2D6 on metoprolol pharmacokinetics and pharmacodynamics. Clin Pharmacol Ther. 2004 Oct;76(4):302–12.

3. Högstedt S, Lindberg B, Peng DR, Regårdh CG, Rane A. Pregnancy-induced increase in metoprolol metabolism. Clin Pharmacol Ther. 1985 Jun;37(6):688–92.

4. Kupferschmidt HH, Ha HR, Ziegler WH, Meier PJ, Krähenbühl S. Interaction between grapefruit juice and midazolam in humans. Clin Pharmacol Ther. 1995 Jul;58(1):20–8.

5. Bornemann LD, Min BH, Crews T, Rees MM, Blumenthal HP, Colburn WA, et al. Dose dependent pharmacokinetics of midazolam. Eur J Clin Pharmacol. 1985;29(1):91–5.

6. Hebert MF, Easterling TR, Kirby B, Carr DB, Buchanan ML, Rutherford T, et al. Effects of pregnancy on CYP3A and P-glycoprotein activities as measured by disposition of midazolam and digoxin: a University of Washington specialized center of research study. Clin Pharmacol Ther. 2008 Aug;84(2):248–53.

7. Kanto J, Sjövall S, Erkkola R, Himberg JJ, Kangas L. Placental transfer and maternal midazolam kinetics. Clin Pharmacol Ther. 1983 Jun;33(6):786–91.

8. Mattila J, Männistö PT, Mäntylä R, Nykänen S, Lamminsivu U. Comparative pharmacokinetics of metronidazole and tinidazole as influenced by administration route. Antimicrob Agents Chemother. 1983 May;23(5):721–5.

9. Loft S, Døssing M, Poulsen HE, Sonne J, Olesen KL, Simonsen K, et al. Influence of dose and route of administration on disposition of metronidazole and its major metabolites. Eur J Clin Pharmacol. 1986;30(4):467–73.

10. Amon I, Amon K, Franke G, Mohr C. Pharmacokinetics of Metronidazole in pregnant women. Chemotherapy. 1981;27(2):73–9.

11. Wang X, Nanovskaya TN, Zhan Y, Abdel-Rahman SM, Jasek M, Hankins GDV, et al. Pharmacokinetics of metronidazole in pregnant patients with bacterial vaginosis. J Matern-Fetal Neonatal Med Off J Eur Assoc Perinat Med Fed Asia Ocean Perinat Soc Int Soc Perinat Obstet. 2011 Mar;24(3):444–8.

12. Visser AA, Hundt HK. The pharmacokinetics of a single intravenous dose of metronidazole in pregnant patients. J Antimicrob Chemother. 1984 Mar;13(3):279–83.

13. Heisterberg L. Placental transfer of metronidazole in the first trimester of pregnancy. J Perinat Med. 1984;12(1):43–5.

14. Karhunen M. Placental transfer of metronidazole and tinidazole in early human pregnancy after a single infusion. Br J Clin Pharmacol. 1984 Aug;18(2):254–7.

15. Schoenwald RD, Huang HS. Corneal penetration behavior of beta-blocking agents I: Physiochemical factors. J Pharm Sci. 1983 Nov;72(11):1266–72.

16. Regårdh CG, Borg KO, Johansson R, Johnsson G, Palmer L. Pharmacokinetic studies on the selective beta1-receptor antagonist metoprolol in man. J Pharmacokinet Biopharm. 1974 Aug;2(4):347–64.

17. Lennernäs H. Intestinal permeability and its relevance for absorption and elimination. Xenobiotica Fate Foreign Compd Biol Syst. 2007 Nov;37(10–11):1015–51.

18. Madani S, Paine MF, Lewis L, Thummel KE, Shen DD. Comparison of CYP2D6 content and metoprolol oxidation between microsomes isolated from human livers and small intestines. Pharm Res. 1999 Aug;16(8):1199–205.

19. Midazolam (base) Safety data sheet [Internet]. Available from: https://www.roche.com/dam/jcr:00d46d73-18f0-4208-b3d7-bbab327eba2d/en/0406732.20200214.12557.pdf

20. Andersin R. Solubility and acid-base behaviour of midazolam in media of different pH, studied by ultraviolet spectrophotometry with multicomponent software. J Pharm Biomed Anal. 1991;9(6):451–5.

21. Sjöberg Å, Lutz M, Tannergren C, Wingolf C, Borde A, Ungell AL. Comprehensive study on regional human intestinal permeability and prediction of fraction absorbed of drugs using the Ussing chamber technique. Eur J Pharm Sci Off J Eur Fed Pharm Sci. 2013 Jan 23;48(1–2):166–80.

22. Gertz M, Houston JB, Galetin A. Physiologically based pharmacokinetic modeling of intestinal first-pass metabolism of CYP3A substrates with high intestinal extraction. Drug Metab Dispos Biol Fate Chem. 2011 Sep;39(9):1633–42.

23. de Vries JX, Rudi J, Walter-Sack I, Conradi R. The determination of total and unbound midazolam in human plasma. A comparison of high performance liquid chromatography, gas chromatography and gas chromatography/mass spectrometry. Biomed Chromatogr BMC. 1990 Jan;4(1):28–33.

24. Paine MF, Khalighi M, Fisher JM, Shen DD, Kunze KL, Marsh CL, et al. Characterization of interintestinal and intraintestinal variations in human CYP3A-dependent metabolism. J Pharmacol Exp Ther. 1997 Dec;283(3):1552–62.

25. Anderson RF, Patel KB. Effect of lipophilicity of nitroimidazoles on radiosensitization of hypoxic bacterial cells in vitro. Br J Cancer. 1979 Jun;39(6):705–10.

26. Cho MJ, Kurtz RR, Lewis C, Machkovech SM, Houser DJ. Metronidazole phosphate--a water-soluble prodrug for parenteral solutions of metronidazole. J Pharm Sci. 1982 Apr;71(4):410–4.

27. Sanvordeker DR, Chien YW, Lin TK, Lambert HJ. Binding of metronidazole and its derivatives to plasma proteins: an assessment of drug binding phenomenon. J Pharm Sci. 1975 Nov;64(11):1797–803.

28. Pearce RE, Cohen-Wolkowiez M, Sampson MR, Kearns GL. The role of human cytochrome P450 enzymes in the formation of 2-hydroxymetronidazole: CYP2A6 is the high affinity (low Km) catalyst. Drug Metab Dispos Biol Fate Chem. 2013 Sep;41(9):1686–94.
